# Inhibitory effect of *Phyllanthus urinaria* L. extract on the replication of lamivudine-resistant hepatitis B virus *in vitro*

**DOI:** 10.1186/s12906-015-0792-3

**Published:** 2015-07-29

**Authors:** Jaesung Jung, Nam Keun Kim, Sun Park, Ho-Joon Shin, Seong Gyu Hwang, Kyongmin Kim

**Affiliations:** Department of Microbiology, Ajou University School of Medicine, Woncheon-dong 5, Suwon, 443-721 Korea; Department of Biomedical Science, Graduate School of Ajou University, Woncheon-dong 5, Suwon, Korea; Institute for Clinical Research, CHA Bundang Medical Center, CHA University, Seongnam, Gyeonggi-do Korea; Department of Internal Medicine, CHA Bundang Medical Center, CHA University, Seongnam, Gyeonggi-do Korea

**Keywords:** Phyllanthus extract, Hepatitis B virus DNA synthesis, Lamivudine-resistant HBV

## Abstract

**Background:**

Long-term treatment of chronic hepatitis B (CHB) with nucleos(t)ide analogs results in the emergence of drug-resistant hepatitis B virus (HBV) harboring mutations in the polymerase (P) gene. The *Phyllanthus* extract has anti-HBV activity; however, its antiviral activity against lamivudine (LMV)-resistant mutants has not been examined.

**Methods:**

HBV harboring LMV-resistant mutations (rtM204I, rtM204V, and rtM204S) in the P gene at the YMDD (^203^tyrosine-methionine-aspartate-aspartate^206^) reverse transcriptase (RT) active site were generated and their sensitivity to *Phyllanthus urinaria koreanis* extract examined. Southern blotting and real-time PCR were used to determine the concentration of plant extract required to inhibit HBV DNA synthesis by 50 and 90 % (EC_50_ and EC_90_, respectively). An enzyme-linked immunosorbent assay was used to measure the EC_50_ of HBV surface antigen (HBsAg) and HBV core antigen (HBcAg) secretion, and the 50 % cytotoxic concentration of the extract was measured in a 3-(4,5-dimethylthiazol-2-yl)-2,5-diphenyltetrazolium bromide assay. Real-time RT-PCR was used to measure mRNA expression levels.

**Results:**

The expression of intracellular HBV DNAs in HBV WT- or mutant-transfected HepG2 cells decreased upon treatment with *Phyllanthus* extract. The secretion of HBsAg and HBcAg also fell in a dose-dependent manner. *Phyllanthus* extract induced interferon-beta (IFN-β), cyclooxygenase-2 (COX-2), and interleukin-6 (IL-6) mRNA expression in HBV WT-transfected HepG2 cells, possibly via activation of extracellular signal-regulated kinases and c-jun N-terminal kinases and the induction of retinoic acid inducible gene-I, toll-like receptor 3, myeloid differentiation primary response gene 88, and/or tumor necrosis factor receptor-associated factor 6 gene expression. HBV transfection in the absence of extract or exposure of cells to extract alone did not trigger these signaling cascades.

**Conclusions:**

*Phyllanthus* extract inhibited HBV DNA synthesis and HBsAg and HBcAg secretion by replicating cells harboring HBV wild-type and LMV-resistant mutants, likely by inducing the expression of IFN-β, COX-2, and IL-6. These data indicate that *Phyllanthus* extract may be useful as an alternative therapeutic agent for the treatment of drug-resistant CHB patients.

**Electronic supplementary material:**

The online version of this article (doi:10.1186/s12906-015-0792-3) contains supplementary material, which is available to authorized users.

## Background

More than 2 billion people worldwide are infected with the hepatitis B virus (HBV), of whom 400 million are chronically infected. A significant fraction of chronically infected individuals develop liver cirrhosis and hepatocellular carcinoma [1, 2], and approximately 1 million die annually from HBV-induced liver disease [3]. HBV is a small, enveloped DNA virus belonging to the *Hepadnaviridae* family, which replicates preferentially in liver cells and utilizes a unique replication strategy involving the reverse transcription of pregenomic RNA (pgRNA) [4].

Interferons (IFNs) and several nucleos(t)ide analogs including lamivudine (LMV), adefovir dipivoxil (ADV), entecavir (ETV), tenofovir (TDF), and telbivudine (LdT), are approved for the treatment of chronic hepatitis B (CHB) in most countries [5]. The low response rate and side effects associated with IFNs mean that nucleos(t)ide analogs are used most often. Nucleos(t)ide analogs inhibit the reverse transcriptase (RT) activity of the HBV DNA polymerase (P), thereby interrupting the elongation of newly synthesized DNA. The drugs also lower alanine aminotransferase levels, reduce serum HBV DNA levels, and induce seroconversion of HBV e antigen (HBeAg) to anti-HBe in CHB patients.

LMV was the first nucleoside analog to be licensed (in 1998); however, 80 % of patients become resistant after 5 years of therapy [5-8]. The most common mutation associated with LMV resistance is the substitution of methionine 204 with isoleucine, valine, or serine (M204I/V/S) at the YMDD active site motif within the P protein RT domain [9–11]. The L80V/I mutation was first detected in patients with severe hepatitis after apparent LMV failure [12]. Mutation at the L180 or A181 residues also contributes to LMV resistance [13]. M204V/I mutations associated with LMV and LdT resistance also confer cross-resistance to other L-nucleosides and reduce sensitivity to ETV (but not to ADV or TDF) [10, 13–17]. ADV-resistance also becomes a limiting factor for treatment, although it develops more slowly than resistance to LMV [18]. Like ADV, TDF (a methyl derivative of ADV) exhibits antiviral activity against LMV-resistant HBV [19], although it is more potent than ADV and the rate of emergence of resistant mutants is slower. However, high doses of TDF and ADV cause nephrotoxicity in CHB patients [8]. Also, because nucleos(t)ide analogs do not prevent the initial formation of covalently closed circular DNA, new cells can be infected during therapy due to the persistent viremia [20].

Several molecules, such as phenylpropenamide derivatives (AT-61 and AT-130) and heteroaryl-pyrimidines (HAP), have been developed to circumvent drug-resistant CHB. Phenylpropenamide derivatives inhibit encapsidation of HBV wild-type (WT) and LMV-resistant mutant pgRNA *in vitro* [21, 22]; however, clinical trials were discontinued due to toxicity [23]. A Phase I clinical trial of HAP, which inhibits HBV core (C) protein dimerization and blocks nucleocapsid formation, has been conducted [24, 25] and both RNAi [26, 27] and inhibitors of a newly identified HBV receptor, sodium taurocholate polypeptide, are being tested [28].

In Asian countries, traditional medicinal herbal extracts have been used to treat chronic liver disease for thousands of years, and modern technology has confirmed their efficacy. Since herbal extracts have been used by millions of people over thousands of years, their safety and low toxicity are strong assets. Several studies describe the anti-HBV activity of traditional herbal extracts. For example, chlorogenic acid, quinic acid, and caffeic acid from the leaves and fruits of the coffee plant [29], *Curcuma longa* Linn extract [30], *Jasminum officinale* L. var. *grandiflorum* [31], emodin (1,3,8-tri-hydroxy-6-methylanthraquinone) [32], oxymatrine from *Sophora radix* [33], and wogonin from *Scutellaria baicalensis* Georgi [34] all suppress HBV replication *in vitro* and/or *in vivo*.

*Phyllanthus* species (*Phyllanthaceae* family) have been used to treat a number of diseases, including human bone disorders [35] and diabetes [36], and show antiviral activity against human immunodeficiency virus [37] and HBV [38–41]. *Phyllanthus* inhibits the activity of hepadnaviral P protein, the secretion HBsAg [38], and transcription of mRNA for the surface (S) protein by specifically interacting with HBV enhancer I [39] and the pre-S1 promoter [40]. It also inhibits viral entry, viral assembly, or virion release by inducing the expression of annexin A7 [41].

The results indicated that *Phyllanthus urinaria koreanis* extract inhibits HBV DNA synthesis and HBsAg and HBcAg secretion by LMV-resistant HBV mutants *in vitro*. It was also evident from the result this did not occur via the inhibition of core particle formation and pgRNA encapsidation. Increased expression of IFN-α, cyclooxygenase-2 (COX-2), and interleukin-6 (IL-6) mRNA by extract-treated HepG2 cells containing replicating HBV may be due to the induction of retinoic acid inducible gene-I (RIG-I), toll-like receptor 3 (TLR-3), myeloid differentiation primary response gene 88 (MyD88), and/or tumor necrosis factor receptor-associated factor 6 (TRAF-6) expression. Taken together, we suggest that *Phyllanthus* extract inhibits HBV replication in HBV WT and LMV-resistant-infected HepG2 cells via the COX-2 and IL-6 signaling pathways.

## Methods

### Preparation of *Phyllanthus urinaria koreanis* aqueous extract

**Fig. 1:**
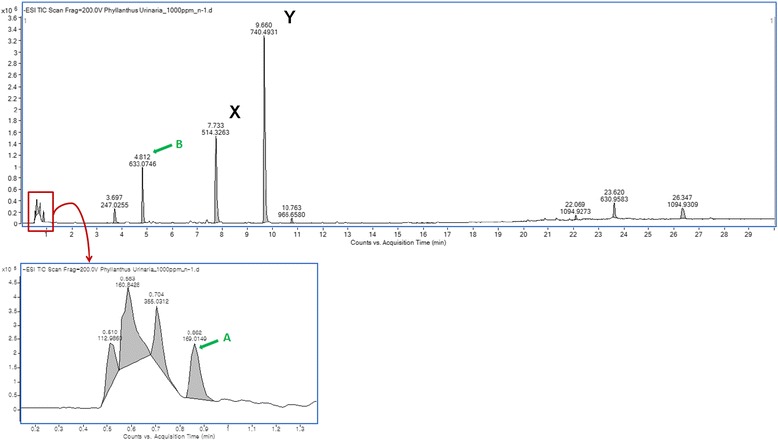
Liquid chromatography/quadruple time-of-flight mass spectrometry analysis of *Phyllanthus urinaria koreanis* extract. A: Gallic acid (2.48 %), B: Corilagin (12.42 %), X and Y: unidentified compounds

*Phyllanthus urinaria koreanis* was provided by Hepaguard Research Laboratories Co. A dried leaf of *Phyllanthus* was ground up and then extracted with water according to the method described by Shin et al. [42]. The aqueous extract was then dried to a powder, dissolved in phosphate buffered saline (final concentration, 100 mg/mL), and stored at −20 °C until required. Skin Biotechnology Center of Kyung Hee University determined chemical composition of the extract by liquid chromatography/quadruple time-of-flight mass spectrometry (LC-qTOF-MS) (Fig. 1).

### Construction of HBV P protein YMDD motif mutants

The full-length monomeric HBV genome was PCR-amplified and subcloned into the *Sac* I/*Sap* I sites in pBluescript SK (Promega) to generate pBluescript-HBV WT. The YMDD (^203^tyrosine-methionine-aspartate-aspartate^206^) RT active site mutants M204A, M204I, M204K, M204L, M204R, M204T, and M204V, were then constructed by PCR-derived mutagenesis. To generate replication competent HBV WT and mutants, the *Eco*R I-*Sac* II fragment from pBluescript-HBV WT and the various YMDD mutants were inserted into pPB, a partially redundant WT HBV subtype adw R9 plasmid [43]. To generate the YSDD variants, Met 204 (ATG) was changed to Ser (**TC**G) using a fusion PCR technique. PCR-derived DNA fragments were generated using the mutagenic primer pairs, 5′-TTT CAG TTA T**TCG**GA TGA TGT GGT ATT G- 3′ and 3′- CA AAC **CGA** AAG TCA ATA AGC CTA CTA CAC- 5′ (mutated sequences in bold and underlined), digested with *Xcm* I-*Sac* II, and inserted into the HBV WT construct to yield the YSDD mutant. All constructs containing PCR-derived DNA fragments were then sequenced to confirm the presence of specific mutations and the absence of extraneous mutations introduced during the PCR reaction.

### Cell culture, transfection, and isolation of core particles

HepG2 cells (a hepatocellular carcinoma cell line) were maintained in Dulbecco’s modified Eagle’s medium (DMEM) (Gibco Life Technologies, Grand Island, NY, USA) supplemented with 10 % heat inactivated fetal bovine serum and 1 % penicillin/streptomycin under a humidified atmosphere at 37 °C containing 5 % CO_2_. Cells were transfected with 8 μg of HBV WT or mutant construct using lipofectamine 2000 (Invitrogen, Carlsbad, CA, USA), according to the manufacturer’s instructions. Cytoplasmic core particles were prepared 3 days post-transfection, as previously described [43]. Transfection experiments were repeated a minimum of three times.

### Southern blotting

To analyze HBV DNA synthesis by Southern blotting, HBV DNA extracted from isolated core particles was separated by agarose gel electrophoresis and hybridized to a ^32^P-labeled random-primed probe specific for the full-length HBV sequence, as described previously [43]. The relative intensities of HBV double-stranded linear (DL) DNA were measured using the Fujifilm Image Gauge V4.0 program (Fuji Film Science lab 2001).

### Western blotting

Isolated core particles were electrophoresed on a 1 % native agarose gel and transferred to polyvinylidene fluoride (PVDF) membrane and then immunoblotted with a polyclonal rabbit anti-HBc antibody (1:1,000; Dako, Glostrup, Denmark), as described previously [43]. Bound antibody was detected with a horseradish-peroxidase (HRP)-conjugated anti-rabbit secondary antibody (Dako) followed by enhanced chemiluminescence (ECL; Amersham, Piscataway, NJ, USA). Total cell lysates were then subjected to SDS-PAGE and the resolved proteins transferred to a PVDF membrane. The membrane was then incubated with monoclonal mouse anti-tubulin (1:1,000; Calbiochem, San Diego, CA, USA), polyclonal rabbit anti-COX-2 (1:1,000; Santa Cruz Biotechnology, Santa Cruz, CA, USA), polyclonal rabbit anti-extracellular signal-regulated kinase (ERK) and anti-p-ERK (1:1,000; Cell Signaling Technology, Danvers, MA, USA), polyclonal rabbit anti-c-jun N-terminal kinase (JNK) and anti-p-JNK (1:1,000 Cell Signaling Technology), polyclonal rabbit anti-p38 and anti-p-p38 (1:1,000 Cell Signaling Technology), or polyclonal rabbit anti-luciferase (1:500; Santa Cruz Biotechnology) antibodies. Immunoreactive bands were visualized using a HRP-conjugated secondary antibody (Dako) followed by ECL. Relative band intensities were measured using the Fujifilm Image Gauge V4.0 program.

### RNase protection analysis (RPA)

An RPA was performed as described previously [43] to analyze the encapsidated and cytoplasmic pgRNA. Radiolabeled anti-sense probes (446 nucleotides (nts); nts 1805–2187 of HBV sequence) were synthesized *in vitro*; the protected sequence comprised 369 nts. The relative levels of cytoplasmic pgRNA and pgRNA obtained from isolated core particles were measured using the Fujifilm Image Gauge V4.0 program.

### Cell cytotoxicity assay

An MTT (3-[4,5-dimethylththiazol-2-yl]-2,5-diphenyltetrazolium bromide) assay was performed to examine the cytotoxic effects of the extracts on HepG2 and Huh7 cells. Cells were grown in 96-well microplates and then incubated with serial dilutions of each extract for 48 h at 37 °C. Cell viability was examined after replacing the culture medium with 100 μL MTT in DMEM. After 3 h, 100 μL dimethyl sulfoxide was added to dissolve formazan of MTT. The absorbance at 570 nm was measured in a plate reader. The CC_50_ (defined as the concentration of extract that reduced cell viability to 50 % of that of the control) was then calculated.

### Quantitative real-time PCR (qPCR) and real-time RT-PCR

HBV DNA extracted from isolated intracellular core particles was analyzed by real-time quantitative PCR in an ABI 7000. The PCR primers (forward primer: HBV, 5′- GAC CAC CAA ATG CCC CTA TC-3′; reverse primer: HBV, 5′- GAG ATT GAG ATC TTC TGC GAC-3′) encompass nts 2301–2443 and overlap the C protein C-terminus and P protein N-terminus. The cycling program was as follows: denaturation at 95 °C for 30 s, followed by 40 cycles of 95 °C for 5 s and 60 °C for 31 s. Each reaction was performed in a well of a 96-well PCR plate in a volume of 20 μL. HBV DNA was quantified using a standard curve. Standard curve was constructed from a serial dilution of HBV WT plasmid with known molecular weight and copy numbers. Total RNA was extracted from cells using RNA isoplus (Takara, Otsu, Shiga, Japan), according to the manufacturer’s instructions. Real-time RT-PCR was then performed to measure the amount of COX-2, IL-6, TNF-α, IFN-β, OAS, PKR, RIG-I, TLR-3, MyD88, and TRAF-6 gene mRNA. Briefly, 5 μg of RNA was reverse transcribed using oligo-dT and the cDNA subjected to quantitative real-time PCR. The primers used for real-time PCR and real-time RT-PCR are listed in Additional file 1.

## Results

### Composition of the *Phyllanthus urinaria koreanis* extract

The amounts of the four major components of *Phyllanthus* extract (corilagin, gallic acid, quercetin, and geraniin) were determined by LC-qTOF-MS, as previously described [44, 45]. In agreement with a previous report [44], corilagin (12.42 %) was detected as a major peak. Gallic acid (2.48 %) was also detected, but the peak was small (Fig. 1). However we could not detect quercetin (MW, 302) or geraniin (MW, 952), and we were unable to identify the other major peaks shown in Fig. 1. From these, we could exclude the possibility that quercetin and geraniin may be responsible for the anti-HBV activity of *Phyllanthus* extract. Since we could not identify the other major peaks as specific compounds, they may therefore play a role in anti-HBV activity.

### *Phyllanthus* extract inhibits HBV DNA synthesis without affecting core particle formation and pgRNA encapsidation

**Fig. 2:**
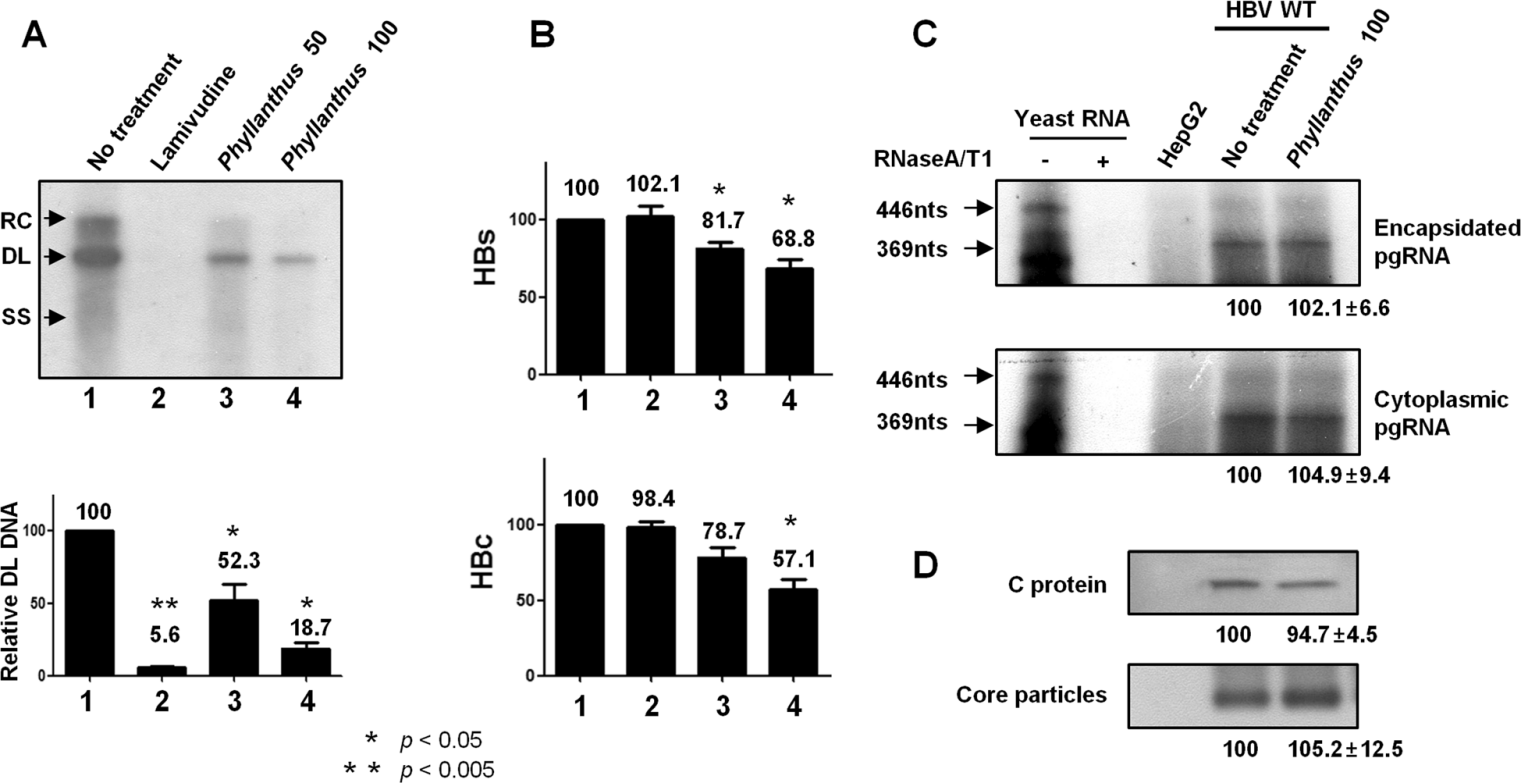
*Phyllanthus urinaria koreanis* extract inhibits HBV DNA synthesis and the secretion of HBsAg and HBcAg. **a** Southern blot analysis to determine HBV DNA synthesis after treatment with LMV or *Phyllanthus* extract. At 1 day post-transfection, HepG2 cells were treated with 50 or 100 μg/mL of *Phyllanthus* extract or LMV (4 μg/mL) for 48 h. HBV DNA was then extracted from isolated core particles, separated, transferred to nylon membranes, hybridized with a random-primed ^32^P-labeled HBV-specific probe, and subjected to autoradiography. Single-stranded and double-stranded linear DNA, and partially double-stranded relaxed circular DNA, are marked as SS, DL, and RC, respectively. The graph shows the level of DL DNA relative to that of HBV DNA in the absence of treatment, as measured by the Fujifilm Image Gauge V4.0 program. **b** *Phyllanthus* extract inhibits the secretion of HBsAg and HBcAg from HBV WT-transfected HepG2 cells. **c** RNase protection analysis of HBV cytoplasmic and encapsidated pgRNA from *Phyllanthus* extract-treated HepG2 cells. The upper panel shows encapsidated pgRNA from isolated core particles, and the lower panel depicts cytoplasmic pgRNA. **d** Western blot analysis to examine the expression of C protein and core particles in *Phyllanthus* extract-treated HepG2 cells. Cell lysates were separated by SDS-PAGE and C protein (*upper panel*) was detected by Western blotting with a rabbit anti-HBc antibody. Isolated core particles were separated on a native agarose gel and analyzed by Western blotting with a rabbit anti-HBc antibody (*lower panel*). The relative levels of pgRNA, C protein, and core particles were measured using the Fujifilm Image Gauge V4.0 program. Statistical significance was evaluated using Student’s *t*-test. *p < 0.05 and **p < 0.005, relative to untreated HBV WT

We next examined HBV C protein expression, core particle formation, pgRNA expression and encapsidation, HBsAg and HBcAg secretion, and DNA synthesis in HBV WT-transfected/*Phyllanthus* extract-treated HepG2 cells (Fig. 2). In agreement with previous reports [38–41], treating cells with different concentrations of extract led to a dose-dependent reduction in intracellular HBV DNA synthesis and in the level of HBsAg and HBcAg secretion (Fig. 2a-b; lanes 3 and 4). Treatment with LMV (4 μg/mL) led to a significant reduction in HBV DNA synthesis (Fig. 2a, lane 2) without affecting the secretion of HBsAg and HBcAg (Fig. 2b, lane 2).

It is not known whether *Phyllanthus* extract affects C protein expression, core particle formation, and pgRNA encapsidation; therefore, we next examined these parameters in HBV WT-transfected/extract-treated HepG2 cells (Fig. 2c-d). Of note, because pgRNA transcription in WT and LMV-resistant mutants was under the control of the cytomegalovirus immediate early (CMV IE) promoter, not the authentic precore/core promoter, pgRNA expression should not be affected by exposure to the extract. Even though the secretion of HBcAg fell upon exposure to the extract, neither C protein expression nor core particle formation was affected (Fig. 2b and d). Also, pgRNA encapsidation was not affected (Fig. 2d). These results suggest that *Phyllanthus* extract inhibits HBV DNA synthesis and the secretion of HBsAg and HBcAg without affecting core particle formation and pgRNA encapsidation.

### Effects of *Phyllanthus* extract on cell viability and HBV replication

Next, we performed an MTT assay to determine the 50 % cytotoxic concentration (CC_50_) of *Phyllanthus* extract for HepG2 cells. The mean CC_50_ of the extract was 757.0 ± 56.5 μg/mL (Additional file 2), which was much higher than that for Huh7 cells (130.1 ± 5.1 μg/mL; Additional file 2).

We next determined the 50 % effective concentration (EC_50_) of *Phyllanthus* extract with respect to HBV WT DNA synthesis in HepG2 cells at 24 h post-transfection for 48 h extract treatment afterward. Southern blotting was then performed to measure the HBV DNA level in HBV WT-transfected/extract-treated HepG2 cells. The levels of double-stranded linear (DL) HBV DNA were quantified using Image Gauge V4.0. The EC_50_ and EC_90_ were 78.6 ± 1.3 and 154.8 ± 11.8 μg/mL, respectively (Table 1). The selectivity index (SI; CC_50_/EC_50_) for HBV WT DNA was 9.63 (Table 1). The EC_50_ of the extract with respect to HBsAg and HBcAg secretion was 252.5 ± 27.0 and 185.9 ± 30.1 μg/mL, respectively (as determined by enzyme-linked immunosorbent assay (ELISA)). The SIs for HBsAg and HBcAg secretion were 2.99 and 4.09, respectively (Table 1). The Southern blotting results were in agreement with those obtained by real-time PCR (Table 1).

**Table 1:** *Phyllanthus urinaria koreanis* extract inhibits replication of HBV WT and LMV-resistant mutants

	HBV DNA by Southern blotting^a^			HBV DNA by qPCR^b^			HBs Ag		HBc Ag	
	EC_50_^c^	EC_90_^d^	SI^e^	EC_50_^c^	EC_90_^d^	SI^e^	EC_50_^c^	SI^e^	EC_50_^c^	SI^e^
HBV WT	78.6 ± 1.3	154.8 ± 11.8	9.63	119.8 ± 21.2	228.2 ± 11.8	6.31	252.5 ± 27.0	2.99	185.9 ± 30.1	4.09
YIDD	82.3 ± 18.6	183.1 ± 22.3	9.19	125.2 ± 18.6	234.7 ± 22.3	6.04	239.5 ± 16.3	3.16	234.4 ± 19.3	3.23
YVDD	104.6 ± 9.2	179.4 ± 32.4	7.23	126.1 ± 9.2	228.2 ± 11.8	6.00	263.7 ± 10.4	2.87	241.3 ± 25.3	3.14
YSDD	90.1 ± 13.2	192.3 ± 22.6	6.91	139.8 ± 33.7	242.6 ± 32.4	5.4	ND	ND	195.6 ± 20.2	3.87

Each value represents the mean ± SD from three independent experiments. HBV DNA was extracted from isolated intracellular core particles after various concentrations of *Phyllanthus urinaria koreanis* extract treatment for 48 h from HBV WT and mutant-transfected HepG2 cells

*ND* not determined

^a^HBV DNA level was measured by Southern blotting using Fujifilm Image Gauge V 4.0 program

^b^HBV DNA level was measured by real-time quantitative PCR. Primers for pRCR are listed in Additional file 1

^c^EC_50_ is the concentration at which 50 % depression of HBV DNA and HBs/e Ag secretion was observed

^d^EC_90_ is the concentration at which 90 % depression of HBV DNA was observed

^e^Selectivity index (SI) was calculated by EC_50_/CC_50_. CC_50_ of extract on HepG2 cells was 757.0 ± 56.5 μg/ml.CC_50_, the drug concentration that reduced cell viability to 50 % of that the control was determined by MTT assay

### Replication of HBV P protein YMDD motif mutants

**Fig. 3:**
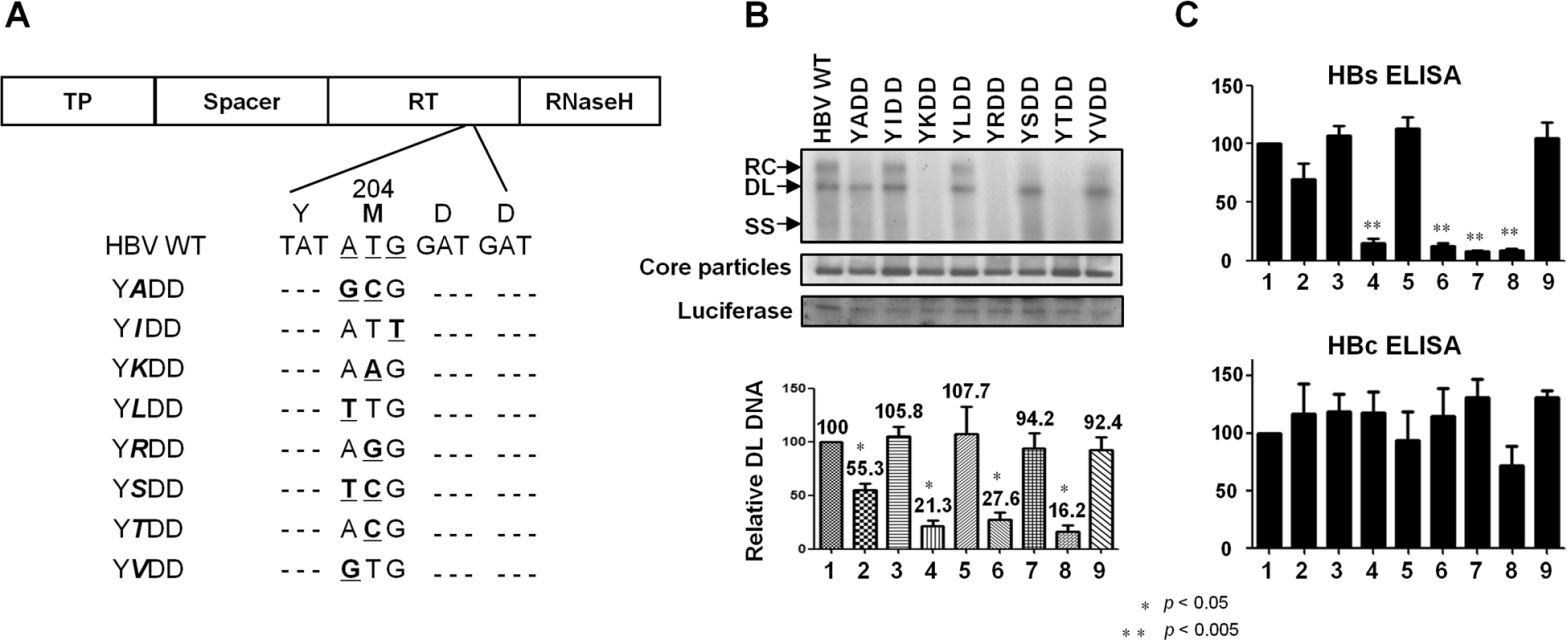
Mutations within the YMDD motif of the HBV P protein, and HBV DNA synthesis, core particle formation, and HBsAg and HBcAg secretion in HBV WT- or YMDD motif mutant-transfected HepG2 cells. **a** Mutated sequences within the YMDD motif mutants. The mutated sequences are indicated by bold underlined letters. The substituted amino acid is indicated in bold italics. Y, tyrosine, M, methionine, D, aspartate, A, alanine; I, isoleucine; K, lysine; L, leucine; R, arginine; S, serine: T, threonine; V, valine. **b** HBV DNA synthesis and core particle formation in HBV WT- and YMDD motif mutant-transfected HepG2 cells. Southern blot and Western blot analyses of HBV DNA synthesis and core particle formation, respectively, in HBV WT- or YMDD motif mutant-transfected HepG2 cells was performed as described in Fig. 2a and d. Each transfection experiment was performed more than three times. Single-stranded and double-stranded linear, and partially double-stranded relaxed circular, DNA are marked as SS, DL, and RC, respectively. Relative levels of HBV DL DNA were quantified using Image Gauge V4.0. (Fuji film), and transfection efficiency normalized according to the level of luciferase expression. **c** ELISA to measure HBsAg or HBcAg secreted from HBV WT- and YMDD motif mutant-transfected HepG2 cells. At 3 days post-transfection, the culture supernatant was collected and the levels of secreted HBsAg and HBcAg were measured in an ELISA. Data are expressed as the mean ± SD of three independent experiments. Statistical significance was evaluated using Student’s *t*-test. *p < 0.05 and **p < 0.005, relative to HBV WT

The conserved ^203^YMDD^206^ motif within the RT catalytic C sub-domain of the HBV P protein is responsible for RNA- and DNA-dependent DNA polymerase activity. M204V or M204I are the most frequently observed mutations in this region, and confer resistance to LMV, LdT, and ETV [16, 17]. A less frequently observed mutation following LMV treatment is M204S, although additional mutations can be found in different RT regions [11]. To investigate the inhibitory effect of *Phyllanthus* extract on LMV-resistant mutants, we substituted methionine 204 with alanine, isoleucine, lysine, leucine, arginine, serine, threonine, or valine to generate YADD, YIDD, YKDD, YLDD, YRDD, YSDD, YTDD, or YVDD, respectively (Fig. 3a). We then examined the replication of these mutants by Southern blot analysis (Fig. 3b, top panel). Single-stranded (SS), DL, and partially double-stranded relaxed circular (RC) HBV DNA molecules were detected in HBV WT- or mutant-transfected cells, albeit with different replication efficiencies (Fig. 3b, top panel). The transfection efficiency was normalized against luciferase expression (Fig. 3b, third panel). The amount of HBV DL DNA isolated from YIDD-, YLDD-, YSDD-, and YVDD-transfected cells was 105.8, 107.7, 94.2, and 92.4 %, respectively, of that isolated from HBV WT (Fig. 3b, top and bottom panels). However, the amount of HBV DL DNA in YADD-transfected cells was 55.3 % of that in HBV WT. HBV DNA synthesis was very inefficient in YKDD-, YRDD-, and YTDD-transfected cells, with HBV DL DNA levels being 21.3, 27.6, and 16.2 % of that in the HBV WT-transfected cells (Fig. 3b, top and bottom panels). Core particles were formed at comparable levels in mutant- and WT-transfected cells (Fig. 3b, 2nd panel).

Next, secretion of HBsAg and HBcAg was examined by ELISA. Overlap of the HBs open reading frame (ORF) with the P gene leads to an alteration in two amino acids (Ile195 and Trp196) in all mutants (except YLDD); this change is likely to affect HBsAg secretion. In agreement with a previous report [9], we observed reduced HBsAg secretion in cells transfected with YADD, YKDD, or YTDD (Fig. 3c). The level of HBsAg secretion from YSDD-transfected cells was low because the YSDD mutant harbored an AT**T C**GG sequence in the HBs ORF, which codes Ile195 and Arg196 (the same as the HBs-negative YKDD and YTDD mutants) (Fig. 3c). It should be noted here that the YSDD mutation identified in a LMV-resistant patient harbored A**GT**, coding for Ile195 and Val196 in the HBs ORF, and was HBsAg positive [11], whereas our constructed mutant harbored **TC**G. In contrast to a previous report [9], we found that the YRDD mutant secreted a very low level of HBsAg and replicated inefficiently (Fig. 3c). With the exception of the YTDD mutant, HBcAg secretion by the mutant viruses was comparable with that by HBV WT (Fig. 3c).

### Anti-HBV activity of *Phyllanthus* extract against the YMDD motif mutants

**Fig. 4:**
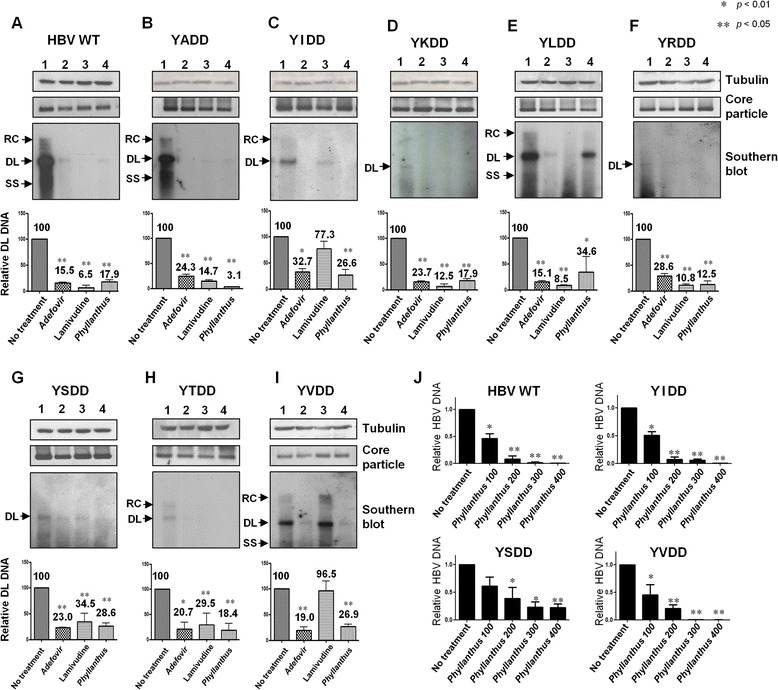
*Phyllanthus urinaria koreanis* extract inhibits replication of YMDD motif mutants. Southern blot analysis to examine the inhibitory effect of *Phyllanthus* extract on **a** HBV WT- or **b**–**i** mutant-transfected HepG2 cells. HBV WT*-* or YMDD motif mutant-transfected cells were mock-treated (*lane 1*), or treated with ADV (100 μM, lane 2), LMV (4 μg/mL, lane 3), or *Phyllanthus* extract (100 μg/mL, lane 4) for 48 h. HBV DNA was then extracted from isolated core particles and analyzed by Southern blotting. ADV was used as a positive control. Core particles were detected as described in Fig. 2d. α-tubulin was used as a loading control. Graphs show the relative levels of HBV DL DNA in HBV WT- or YMDD motif mutant-transfected HepG2 cells after 48 h exposure to extract, LMV, or ADV (as measured by Image Gauge V4.0; Fuji film). Statistical significance was evaluated using Student’s *t*-test. *p < 0.05 and **p < 0.005, relative to the respective HBV WT or mutant-transfected cells not treated with extract. **j** Amounts of core particles associated with HBV DNA as assessed by quantitative real-time PCR. HBV DNA was extracted from core particles isolated from HBV WT- and LMV-resistant YIDD-, YSDD-, and YVDD-mutant-transfected HepG2 cells after 48 h exposure to different concentrations of *Phyllanthus* extract. *p < 0.005 and **p < 0.0005, relative to the respective HBV WT- or mutant-transfected but untreated cells

HepG2 cells were transfected with HBV WT or YMDD motif mutants. Twenty-four hours later, the cells were exposed to *Phyllanthus* extract (100 μg/mL) for 48 h. Replicative intermediate HBV DNAs isolated from intracellular core particles were then analyzed by Southern blot analysis (Fig. 4). Cells were treated with LMV (4 μg/mL) and ADV (27.3 μg/mL or 100 μM) as controls (Fig. 4, lanes 2 and 3). HBV DL DNA synthesis in LMV-, ADV-, and *Phyllanthus* extract-treated HBV WT-transfected HepG2 cells was 6.5, 15.5, and 17.9 % of that in untreated WT-transfected HepG2 cells (Fig. 4a). Similarly, LMV, ADV, and *Phyllanthus* extract inhibited HBV DNA synthesis in YADD-, YKDD-, YRDD- and YTDD-transfected HepG2 cells (Fig. 4b, d, f, and h). Consistent with previous reports, HBV DNA synthesis by YIDD and YVDD mutants was not inhibited by LMV treatment, confirming that these mutants were LMV-resistant (Fig. 4c and i, lanes 3). However, HBV DNA synthesis by the YIDD and YVDD mutants was inhibited by ADV and *Phyllanthus* extract (Fig. 4c and i, lanes 2 and 4). This, *Phyllanthus* extract maybe a useful treatment for LMV-resistant CHB patients. A previous study demonstrated that the YSDD variant was LMV-resistant [11]; however, we found that HBV DNA synthesis by the YSDD mutant was only marginally inhibited by LMV (34.5 % HBV DL DNA) (Fig. 4g, lane 3), indicating that an additional L180M mutation in the P gene [11] might confer LMV resistance. Nevertheless, ADV and *Phyllanthus* extract inhibited HBV DNA synthesis by the YSDD mutant more efficiently than LMV (Fig. 4g).

The EC_50_ and EC_90_ values for the *Phyllanthus* extract against HBV WT and the YIDD, YSDD, and YVDD mutants calculated after Southern blotting (Table 1) and real-time PCR were similar (Table 1, Fig. 4j). The EC_50_ for HBsAg and HBcAg secretion by the mutants were also determined by ELISA (Table 1), and showed that the SIs for the mutants were comparable with those for the WT. We could not determine the level of HBsAg in YSDD-transfected cells because they secreted low levels of HBsAg. Taken together, these results demonstrate that *Phyllanthus* extract inhibits both HBV DNA synthesis and HBsAg and HBcAg secretion by LMV-resistant mutants.

### *Phyllanthus* extract triggers intracellular signaling and innate immune responses in HBV replicating HepG2 cells

**Fig. 5:**
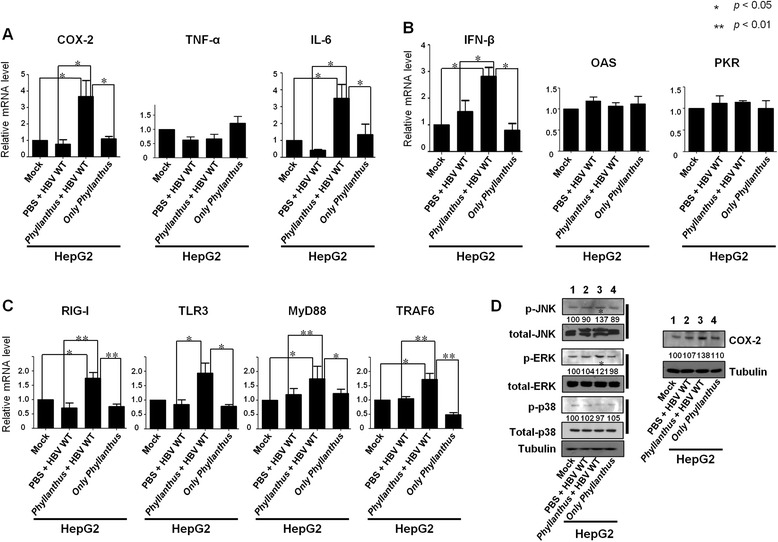
Effects of the extract on intracellular signaling. Quantitative real-time RT-PCR was used to determine the levels of **a** COX-2, TNF-α, and IL-6, **b** IFN-β, OAS, and PKR, and **c** RIG-I, TLR-3, MyD88, and TRAF-6 mRNA. Total RNA was isolated from mock-, HBV WT-transfected, HBV WT-transfected/extract-treated (100 μg/mL), or extract-only-treated (100 μg/mL) HepG2 cells at 3 days post-transfection and subjected to real-time RT-PCR. Data represent the mean level of mRNA expression from three independent experiments. Statistical significance was evaluated using Student’s *t*-test. *p < 0.05 and **p < 0.01, relative to the respective mock-, untreated HBV WT, treated HBV WT, or extract-only-treated HepG2 cells. **d** ERK1/2 and JNK activation and COX-2 protein expression in mock-, HBV WT- transfected, HBV WT-transfected/extract-treated (100 μg/mL), or extract-only-treated (100 μg/mL) HepG2 cells

Medicines derived from plants inhibit viral production by inducing inflammatory responses and/or the production of pro-inflammatory cytokines [46, 47]. Here, we observed increased expression of mRNA for COX-2 and the pro-inflammatory cytokine, IL-6 (Fig. 5a). By contrast, there was no increase in the expression of mRNA for TNF-α, another pro-inflammatory cytokine (Fig. 5a). Although expression of IFN-β mRNA increased in HBV WT-transfected/extract-treated cells, there was no change in the expression of mRNA for IFN-inducible genes such as OAS and PKR (Fig. 5b), suggesting that IFN may not exert antiviral activity via transcriptional upregulation of IFN-inducible genes in this HepG2-transfected cell system. The significant reduction in HBV replication and the increased expression of IL-6 and COX-2 in HBV WT-transfected/*Phyllanthus* extract-treated cells led us to hypothesize that upregulation of COX-2 and IL-6 mRNA occurs through transcriptional activation of innate immune signaling genes. We found that the expression of RIG-I, TLR-3, MyD88, and TRAF-6 mRNA increased in HBV WT-transfected/*Phyllanthus* extract-treated cells, but not in HBV WT-transfected cells or in cells treated with the extract alone (Fig 5c). This is suggestive of transcriptional activations of innate immune signaling genes. Also, phosphorylation of ERK1/2 and JNK and expression of COX-2 increased in HBV WT-transfected/extract-treated cells, but not in HBV WT-transfected cells or in cells treated with extract alone (Fig 5d). These results suggest that IL-6 upregulation in HBV-transfected/extract-treated HepG2 cells is mediated by both increased COX-2 expression (via ERK1/2 and JNK activation) and increased RIG-I, TLR-3, MyD88, and TRAF-6 expression. Taken together, these data suggest that *Phyllanthus* extract inhibits HBV replication in HepG2 cells by activating the innate immune response by triggering intracellular signaling cascades (e.g., the ERK1/2 and JNK pathways), leading to the induction of COX-2 and IL-6.

## Discussion

The *Phyllanthus* species has long been utilized as an herbal medicine, including as an anti-HBV agent. Here, we demonstrated that *Phyllanthus* extract has antiviral activity against LMV-resistant HBV mutants (Fig. 4 and Table 1). We also showed that the antiviral activity of *Phyllanthus* extract was not due to inhibition of intracellular HBV RNA and C protein expression, core particle formation, or pgRNA encapsidation (Fig. 2). Therefore, to examine the mechanism underlying *Phyllanthus*-mediated inhibition of HBV DNA replication (Figs. 2 and 4), we analyzed the expression of intracellular signaling molecules. We found increased expression of TLR-3, RIG-I, TRAF-6, and MyD88, COX-2, IL-6, and IFN-β in HepG2 cells transfected with WT HBV and then exposed to *Phyllanthus* extract, but not in HBV WT-transfected HepG2 cells or in HepG2 cells exposed to extract alone (Fig. 5c). This suggests that the extract triggers the innate immune response and/or inflammatory responses, thereby inhibiting HBV replication.

Upregulated IFN-β expression (Fig. 5b) suggests that *Phyllanthus* might induce IFN-mediated antiviral responses via IFN-inducible OAS and PKR genes. However, we did not observe OAS and PKR upregulation in HBV WT-transfected/extract-treated HepG2 cells (Fig. 5b). Since HepG2 cells harbor inherent defects in IFN-mediated antiviral responses [48], and IFN-α/β only modestly inhibits HBV replication in HepG2 cells [49], it appears that IFN-mediated responses may not be the major mechanism underlying the antiviral activity of the extract. However, we cannot exclude the possibility that other IFN-inducible cellular factors, such as apolipoprotein B mRNA-editing enzyme catalysis polypeptide 1-like 3G (APOBEC3G) or human cytidine deaminase may be involved in limiting HBV replication without affecting pgRNA packaging [50]. IFN-α-inducible expression of APOBEC3G has been reported in human hepatocytes, and IFN-responsive elements in the APOBEC3G promoter have been identified [50]. In addition, the DDX3 DEAD box (an RNA helicase) is involved in augmenting IFN-regulatory factor signaling by interacting with TANK-binding kinase 1/IκB kinase ε [51]. Several studies demonstrate that cellular factors, such as APOBEC3G and DDX3, are incorporated into core particles and interact with HBV P protein, thereby inhibiting reverse transcription [52, 53].

The HBV X protein (HBx) and HIV gp120 stimulate COX-2 via the Ras-Raf-mitogen-activated protein kinase cascade, JNK, nuclear factor-κB, and the Janus kinase/signal transducers and activators of transcription signaling pathways [54–57]. Unlike HBx over-expressed cells [55], we found that COX-2 expression was not increased in HBV WT-transfected cells (Fig. 5a and d, lanes 2). Since upregulated expression of COX-2 mRNA and protein was observed only in HBV WT-transfected/extract-treated HepG2 cells (Fig. 5a and d, lanes 3), expression of COX-2 and IL-6 may be triggered in HBV replicating cells in the presence of *Phyllanthus* extract. Upregulation of COX-2 expression via activation of the ERK1/2 and/or JNK pathways may play a role in the observed antiviral activity (Fig. 5d, lane 3). IL-6 regulates early HBV gene expression and inhibits HBV replication by activating the ERK1/2 and JNK pathways [58]. We speculate that increased IFN-β, IL-6, and COX-2 expression in HBV WT-transfected*/Phyllanthus* extract-treated HepG2 cells may induce the expression of IFN-inducible genes and/or different inflammatory mediators, which then inhibit HBV replication.

Taken together, the data presented herein suggest three possible mechanisms underlying the anti-HBV activity of *Phyllanthus* extract: (i) *Phyllanthus* extract directly inhibits hepadnaviral P protein [38]; (ii) because *Phyllanthus* extract alone did not induce a signaling cascade in HepG2 cells (Fig. 5), *Phyllanthus* extract works in conjunction with HBV replication and/or HBV protein and RNA expression to inhibit HBV replication by inducing IFN-β, COX-2, and IL-6 expression; (iii) since intracellular HBV RNAs, proteins, and core particles were unaffected by *Phyllanthus* extract (Fig. 2c-d), these molecules might stimulate the innate immune response to further inhibit HBV replication. These hypotheses are not mutually exclusive and they may work in concert with each other.

## Conclusion

The results presented herein demonstrate that *Phyllanthus* extract effectively inhibits the replication of LMV-resistant HBV. *Phyllanthus* extract appears to inhibit HBV replication by inducing the expression of IFN-β, COX-2, and IL-6, which in turn activate the innate immune response. Thus, *Phyllanthus* extract may be a therapeutic agent useful for the management of LMV-resistant CHB patients; However, the detailed antiviral mechanisms and *in vivo* efficacy require further investigation.

## Additional files

Additional file 1:
**Primers for realtime RT-PCR or PCR.** (TIFF 1258 kb) Additional file 2:
**MTT assay to measure the cytotoxicity of** ***Phyllanthus urinaria koreanis*** **extract.** HepG2 and Huh7 cells were exposed to varying concentrations of *Phyllanthus* extract (50–1200 μg/mL) for 48 h and CC_50_ values determined in an MTT assay. The CC_50_ was defined as the concentration of extract that reduced cell viability to 50 % of that of the control (cells untreated by extract). (TIFF 1052 kb)

## Abbreviations

HBV: Hepatitis B virus
IFN-β: Interferon-beta
COX-2: Cyclooxygenase-2
IL-6: Interleukin-6
LMV: Lamivudine
HBsAg: HBV surface antigen
HBcAg: HBV core antigen
pgRNA: Pregenomic RNA
