# Supplementary figures and images for: Inhibitory effect of Phyllanthus urinaria L. extract on the replication of lamivudine-resistant hepatitis B virus in vitro
Source: BMC Complement Altern Med. 2015 Jul 29;15:255. doi: 10.1186/s12906-015-0792-3 (PMC4518506; doi:10.1186/s12906-015-0792-3)

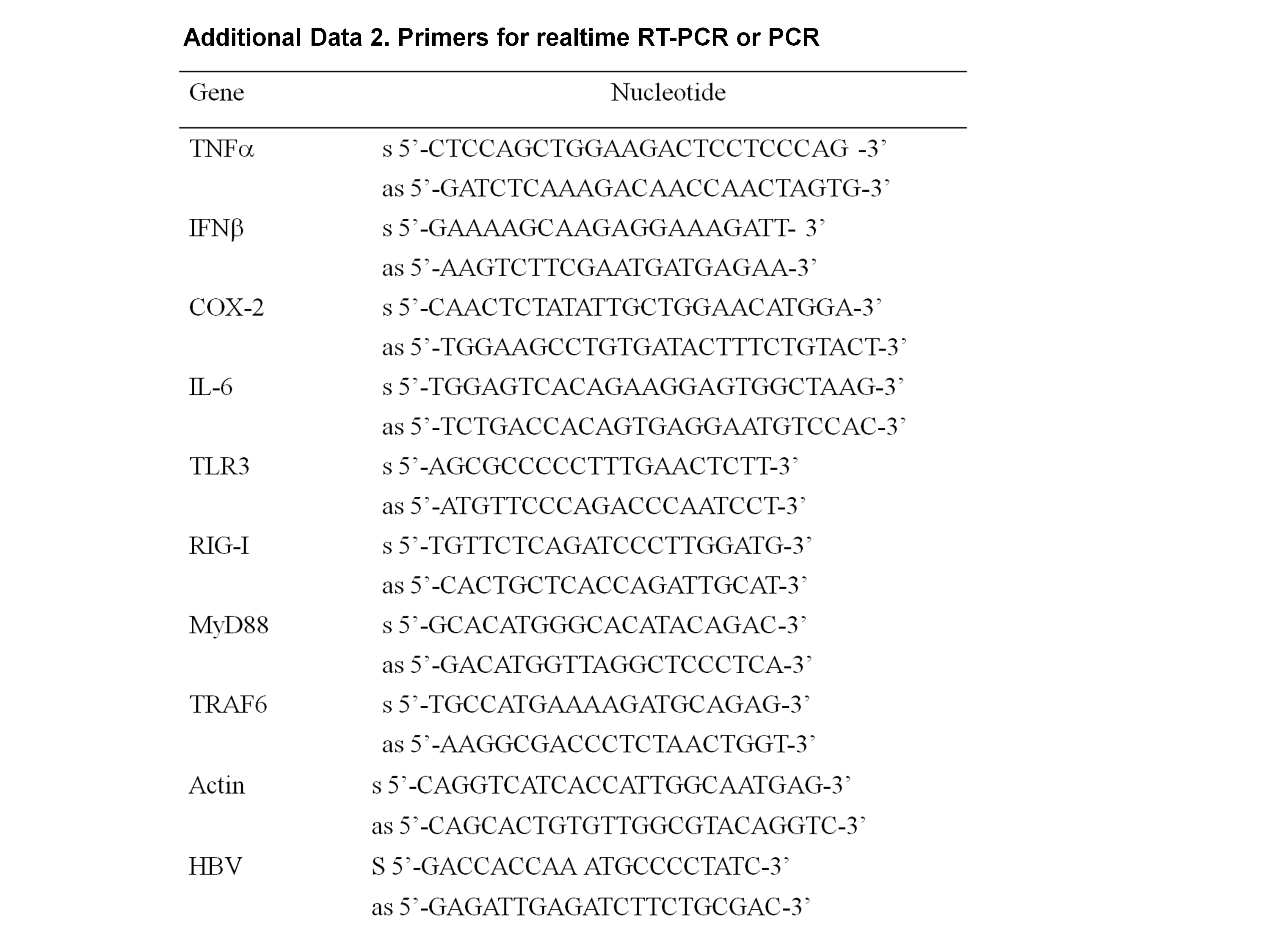

Supplement: Additional file 1: — Primers for realtime RT-PCR or PCR. (TIFF 1258 kb) [file 12906_2015_792_MOESM1_ESM.tiff]

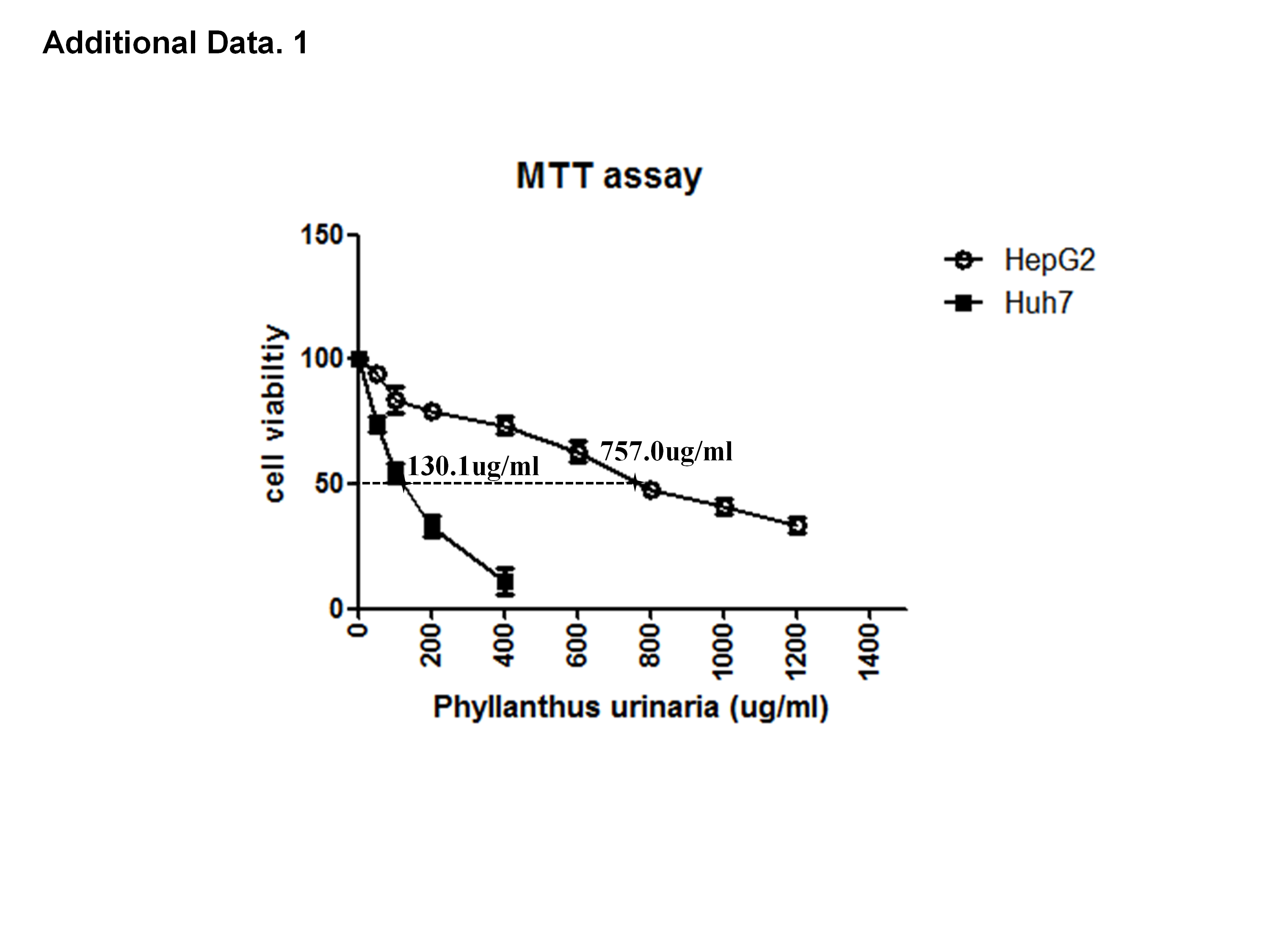

Supplement: Additional file 2: — MTT assay to measure the cytotoxicity of Phyllanthus urinaria koreanis extract. HepG2 and Huh7 cells were exposed to varying concentrations of Phyllanthus extract (50–1200 μg/mL) for 48 h and CC50 values determined in an MTT assay. The CC50 was defined as the concentration of extract that reduced cell viability to 50 % of that of the control (cells untreated by extract). (TIFF 1052 kb) [file 12906_2015_792_MOESM2_ESM.tiff]
